# Expanding SPG18 clinical spectrum: autosomal dominant mutation causes complicated hereditary spastic paraplegia in a large family

**DOI:** 10.1007/s10072-024-07500-0

**Published:** 2024-04-12

**Authors:** Assunta Trinchillo, Valeria Valente, Marcello Esposito, Miriana Migliaccio, Aniello Iovino, Michele Picciocchi, Nunzia Cuomo, Carmela Caccavale, Cristofaro Nocerino, Laura De Rosa, Elena Salvatore, Giovanna Maria Pierantoni, Valeria Menchise, Simona Paladino, Chiara Criscuolo

**Affiliations:** 1 Department of Neurosciences, Reproductive Sciences and Odontostomatology, University Federico II of Naples, Naples, Italy; 2 Department of Molecular Medicine and Medical Biotechnology, University of Naples Federico II, Naples, Italy; 3 Clinical Neurophysiology Unit, Cardarelli Hospital, Naples, Italy; 4 IRCCS SDN SYNLAB, Naples, Italy; 5 CDCD Neurology, “Federico II” University Hospital, Naples, Italy; 6 Institute of Biostructure and Bioimaging, National Research Council (CNR) and Molecular Biotechnology Center, Turin, Italy

**Keywords:** Hereditary spastic paraplegia (HSP), Amyotrophic lateral sclerosis (ALS), ERLIN2, SPG18

## Abstract

**Background:**

SPG18 is caused by mutations in the endoplasmic reticulum lipid raft associated 2 (*ERLIN2*) gene. Autosomal recessive (AR) mutations are usually associated with complicated hereditary spastic paraplegia (HSP), while autosomal dominant (AD) mutations use to cause pure SPG18.

**Aim:**

To define the variegate clinical spectrum of the SPG18 and to evaluate a dominant negative effect of erlin2 (encoded by *ERLIN2*) on oligomerization as causing differences between AR and AD phenotypes.

**Methods:**

In a four-generation pedigree with an AD pattern, a spastic paraplegia multigene panel test was performed. Oligomerization of erlin2 was analyzed with velocity gradient assay in fibroblasts of the proband and healthy subjects.

**Results:**

Despite the common p.V168M mutation identified in *ERLIN2*, a phenoconversion to amyotrophic lateral sclerosis (ALS) was observed in the second generation, pure HSP in the third generation, and a complicated form with psychomotor delay and epilepsy in the fourth generation. Erlin2 oligomerization was found to be normal.

**Discussion:**

We report the first AD SPG18 family with a complicated phenotype, and we ruled out a dominant negative effect of V168M on erlin2 oligomerization. Therefore, our data do not support the hypothesis of a relationship between the mode of inheritance and the phenotype, but confirm the multifaceted nature of SPG18 on both genetic and clinical point of view. Clinicians should be aware of the importance of conducting an in-depth clinical evaluation to unmask all the possible manifestations associated to an only apparently pure SPG18 phenotype. We confirm the genotype–phenotype correlation between V168M and ALS emphasizing the value of close follow-up.

## Introduction

Hereditary spastic paraplegias (HSPs) are a group of clinically and genetically heterogeneous neurodegenerative diseases characterized by progressive spasticity and lower limb weakness.

Currently, more than 80 causal genes or loci have been identified with all possible patterns of inheritance.

From a clinical point of view, HSPs are classified as pure, when presenting just spastic paraplegia, and complicated, when associated with other clinical features as ataxia, seizures, cognitive decline, extrapyramidal symptoms, and peripheral neuropathy.

Mutations in the endoplasmic reticulum lipid raft associated 2 (*ERLIN2*, MIM #611,605) gene are responsible for spastic paraplegia type 18 (SPG18).

The first reported families presented an autosomal recessive (AR) transmission and a complicated phenotype characterized by intellectual disability, joint contractures and deformities, lower limbs spasticity, and weakness [1].

Over the following years, several novel mutations have been detected, expanding genetic spectrum to autosomal dominant (AD) forms mainly associated to pure HSP phenotype [2–5].

Dominant and recessive mutations have also been associated to amyotrophic lateral sclerosis (ALS) [3, 6].

Here, we report the identification of a heterozygous *ERLIN2* variant (c.502G > A) in an extensive SPG18 family from Southern Italy, with a complicated phenotype evolving to ALS. We also analyze the pattern of oligomerization of erlin2 in order to point out a possible dominant-negative effect.

## Patients and methods

### Patients

A four-generation family with HSP was recruited at the Department of Neuroscience of the Federico II University in Naples.

**Fig. 1:**
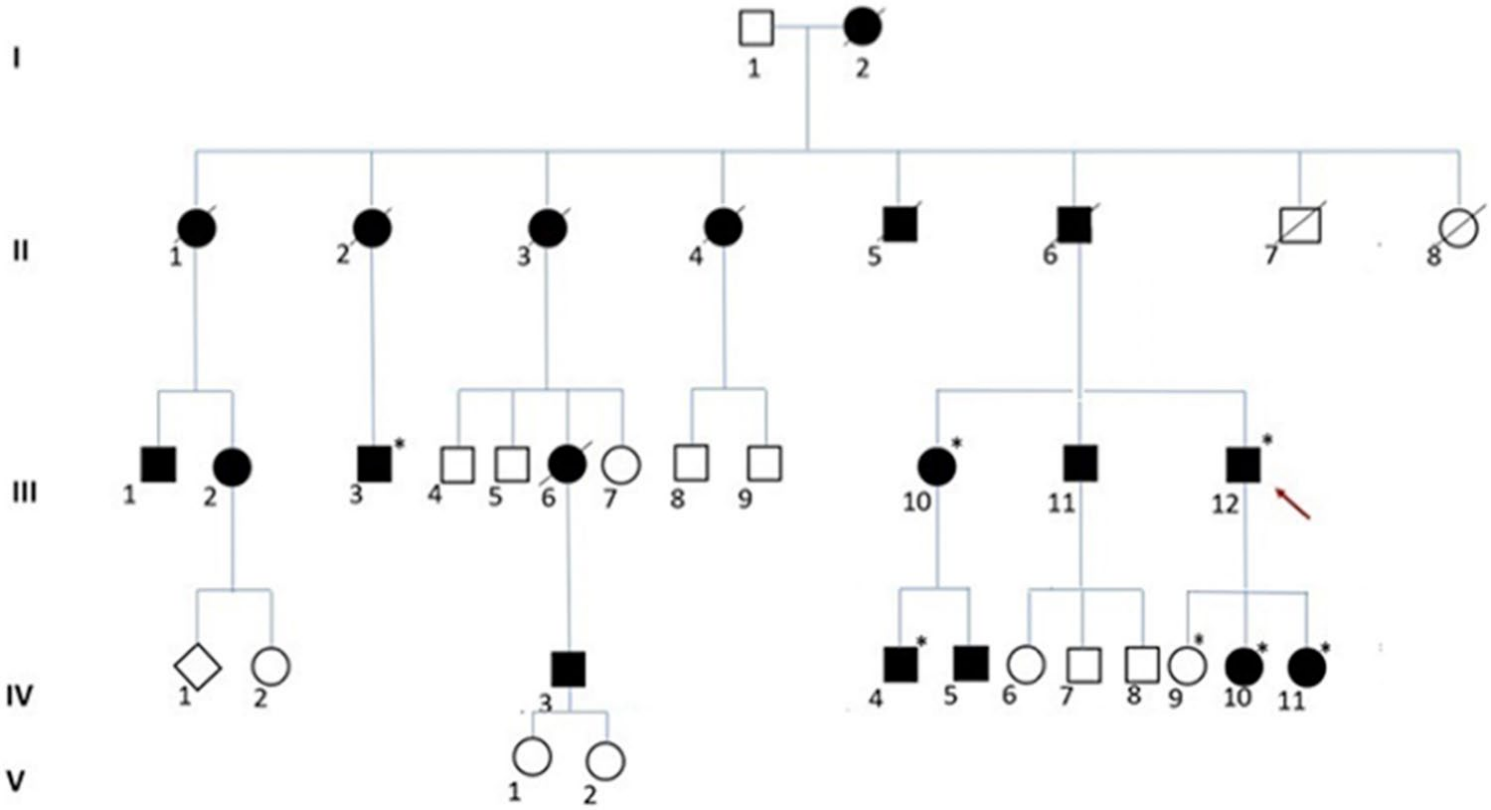
Family pedigree. Black filled symbol affected; white symbol, unaffected; diamond shaped symbol, masked gender. Roman numerals represent the generation. Arabic numerals identify individuals. The arrow indicates the proband. Sanger sequencing was performed in subjects with asterisks: all carried the V168M mutation at heterozygous state except for IV-9

We were able to collect data of seven members [three females (III-11, IV-10, IV-11) and four males (III-12, III-10, III-3, IV-4)] among 19 affected individuals, since all the members of the first and the second generation were deceased before the proband’s (III-12) first neurological referral (Fig. 1).

Therefore, at the time of sampling, first and second generations clinical information was obtained from medical records and from their living relatives.

After informed consent was obtained, a detailed clinical evaluation of III-3, III-12, IV-10, IV-11, and IV-4 was performed by expert neurologists (CC and AT). Peripheral blood samples were collected for genetic analysis from five affected (III-3, III-10, III-12, IV-10, IV-11) and four healthy subjects: the proband’s first daughter (IV-9), his mother, and his two wives (not shown in Fig. 1).

Electromyography (EMG), electroneurography (ENG), and somatosensory and motor evoked potentials (SEPs, MEPs) were performed in cases III-3, III-12, and IV-10; magnetic resonance (MRI) was performed in III-3, III-12, and IV-11 and electroencephalogram (EEG) in III-12, IV-10, and IV-11.

An extensive neuropsychological battery was performed in III-3 and III-12; the Wechsler Intelligence Scale for Children (WISC-V) was administrated to IV-10 and IV-11.

Both siblings of the proband (III-10, III-11) suffered for gait impairments since their 20 s; nevertheless, they refused any kind of clinical/instrumental evaluation.

Patient III-10 accepted to perform only the genetic analysis since her son was affected (IV-4).

### Genetic analysis

Patients III-12 and IV-11 underwent spastic paraplegia multigene panel testing using methodologies and a bioinformatic pipelines already reported elsewhere [7].

Cosegregation between the mutation and the phenotype was evaluated by Sanger sequencing in all family members whose DNA was available.

### Cell cultures and protein activity

Fibroblasts from III-12 were obtained from cultured skin punch biopsies. Written informed consent was obtained from all the patients.

As controls, we used fibroblast cell lines derived from two healthy, age-/gender-matched individuals. Those cells were obtained from our institutional biobank. All cells were investigated at similar culture passages (P4–P6).

Cells were grown in Dulbecco’s modified Eagle’s medium (DMEM, Merck Life Science) supplemented with 2 mM glutamine, 20% FBS (Thermo Fisher Scientific), and penicillin/streptomycin, at 37 °C and 5% CO_2_.

Oligomerization state of erlin2 in fibroblasts from healthy subjects and patient III-12 was evaluated (i) by sedimentation on velocity gradients, a technique in which proteins sediment according to their molecular weight [8], and (ii) by Blue native PAGE (BN-PAGE), an electrophoretic method in which protein complexes are separated in native conditions in acrylamide gradient gels according to their size [9].

Erlin mutant structure has been predicted on the web portal Phyre2 [10] using as template the bacterial HflC protein structure (pdb code: 7VHQ).

## Results

### Patients

The family in the current study comes from Puglia, a region of Southern Italy. Pedigree analysis suggested AD mode of inheritance (Fig. 1).

The proband’s father (II-6) was diagnosed with ALS according to El Escorial criteria, when he was 51 years old. Onset was at 26 years old with gait difficulties. He needed a cane to walk since he was 48 years old, and he died at the age of 54.

The aunt (II-2) was wheelchair-bounded since her 40 s and received the diagnosis of ALS when she was 56, after she was admitted to hospital for respiratory failure. She died 2 years later, for aspiration pneumonia.

Over the last 3 years of their lives they both developed swallowing issues, slurred speech, and breathing difficulties requiring hospitalizations. Four limbs atrophy was reported in their hospital records.

According to the proband and his cousin (III-3), most of the siblings of patient II-6 had similar symptoms: onset with gait impairment in the setting of HSP and ALS-like symptoms in the fifth decade, mainly characterized by swallowing and breathing difficulties, muscular wasting, and premature death.

Detailed clinical features of the proband (III-12), his daughters (IV-10; IV-11), his cousin (case III-3), and his nephew (IV-4) are reported in Table 1.

**Table 1:** Clinical features of the patients with the *ERLIN2* mutation

Patient	III-12—proband	III-3—proband’s cousin	IV-10—proband’s daughter (I marriage)	IV-11—proband’s daughter (II marriage)	IV-4—proband’s nephew
Gender	M	M	F	F	M
Spastic paraplegia age at onset	18	20	10	2	9
Age at examination	38	35	11	6	25
Symptom at onset	LL stiffness	Moderate gait difficulties	LL pain	Walking on toes Speech delay	Speech and motor delay
Horizontal Nystagmus	+	-	-	+	-
Pontobulbar signs	-	-	-	-	-
Spastic gait	+ +	+ + +	+	+ +	+ +
Muscle weakness in UL (MRC scale)	5	5	5	5	5
Muscle weakness in LL^a^ (MRC scale)	4	4	4	5	5
Increased muscle tone in UL	-	-	-	-	-
Increased muscle tone in LL	+ +	+ + +	+	-	+ +
UL and LL atrophy	-	-	-	-	-
Fasciculations	-	-	-	-	-
Hyperreflexia in UL	+	-	-	-	-
Hyperreflexia in LL	+ + +	+ + +	+ + +	+ + +	+ + +
Ankle clonus	+ / + (limited)	+ / + (unlimited)	+ / + (unlimited)	+ / + (limited)	+ / + (limited)
Extensor plantar response	+ / -	-/-	-/-	+ / +	-/-
Bladder Dysfunction	-	-	Urgency	-	-
Reduced vibration sense	-	-	-	-	-
Scoliosis	+	-	+	-	-
Foot deformity	Flat feet	-	Flat feet	Club feet	-
Pain	-	-	+ +	-	+
Seizures	-	-	-	-	+
Psychomotor delay	-	-	+	+	+
Intellectual disability	-	-	+	-	+
SPRS score^b^	18	14	6	6	8
Disability stage^c^	2	2	1	1	2
Additional features	-	Non-Hodgkin lymphoma	-	Varus knees	-

- -, absent; + , mild; +  + , moderate; +  +  + , severe

*UL* upper limbs, *LL* lower limbs, *MRC scale* Medical Research Council

^a^Proximal muscle weakness

^b^SPRS (Spastic Paraplegia Rating Scale) range 0–52

^c^Disability stage (SPATAX-EUROSPA): 0, no functional handicap; 1, no functional handicap but signs at examination; 2, able to run, walking unlimited; 3, unable to run, limited walking without aid; 4, walking with one stick; 5, walking with two sticks; 6, unable to walk; 7, confined to bed (https://spatax.wordpress.com/)

The proband (III-12) underwent a brain and cervical spinal cord MRI showing just one white matter pontine lesion and normal corpus callosum (CC). EMG, ENG, MEPs, and SEPs were unremarkable at the first evaluation and after 2 years of follow-up. EEG showed bifrontal spikes—waves with secondary spread. However, he has never experienced any seizures. An extensive neuropsychological battery [11] performed in patients III-12 and III-3 showed impaired recall of the Rey–Osterrieth Complex Figure test with normal Mini Mental State Examination score (27 and 28, respectively).

EMG, ENG, MEPs, and SEPs and brain MRI were unremarkable in pt. III-3.

The second proband’s daughter (IV-10) was born via C/S because of breech presentation and reached early milestones for sphincter and speech development at an appropriate age, whereas early motor milestones were slightly delayed (20 months).

Due to some adaptive problems, she started school when she was 6 years old and needed some help due to writing and reading issues.

When she was 9 years old, she underwent her first neuropsychological examination showing a mild intellectual impairment. Intellectual quotient (IQ) at the WISC-IV Test (Wechsler Intelligence Scale for Children) was 66, subscores were working memory (WMI) = 67; processing speed of the information (PSI) = 68; perceptive reasoning index (PRI) = 85; verbal comprehension index (VCI) = 76; perceptive reasoning index (PRI) = 85. EMG, ENG, MEPs, and SEPs were all unremarkable at baseline and at a 2-year follow-up.

The last affected daughter (IV-11)—born from the second proband’s marriage—started walking on toes when she was 20 months and presented speech disturbances since the beginning.

Brain MRI was normal with normal CC. An EEG disclosed bilateral occipital spikes and polispikes—waves associated with photoparossistic response to intermittent photic stimulation. Nevertheless, she never experienced clinical seizures, including eyelids myokymia. WISC-IV was in the normal range (IQ = 98) with the following subscores: WMI = 100; PSI = 98; PRI = 100; VCI = 100.

Finally, the nephew of the proband (IV-4), as his two cousins, presented speech and motor delay since he was a child, for which he required significant support during grade school even without any formal diagnosis. His mother, patient III-10, 46 years old, could not walk and had been using a wheelchair for more than 10 years. Opposite to his cousins, his condition has never been investigated until he recognized that some of his disturbances were present in other relatives.

Furthermore, when he was an adolescent, he started to suffer for generalized tonic–clonic seizures, receiving the diagnosis of generalized idiopathic epilepsy which responded to levetiracetam 2000 mg. To date, he is seizure-free thanks to the treatment.

He refused to perform any kind of functional or imaging investigations.

### Genetic analysis

The spastic paraplegia multigene panel testing revealed the heterozygous c.502G > A (p.V168M) in *ERLIN2* in both III-12 and IV-11 patients. It was then confirmed by Sanger sequencing and was found to co-segregates with the disease in the affected patients (III-3, III-10, III-12; IV-4, IV-10, IV-11) while it was not present in the healthy subjects.

Such mutation was already reported in an AD French family with spastic paraplegia converting to ALS [6] and in an AD Chinese kindred with a pure phenotype [12].

Therefore, this is the first report of SPG18 complicated phenotype with AD inheritance related to the mentioned mutation.

### Erlin2 oligomerization

**Fig. 2:**
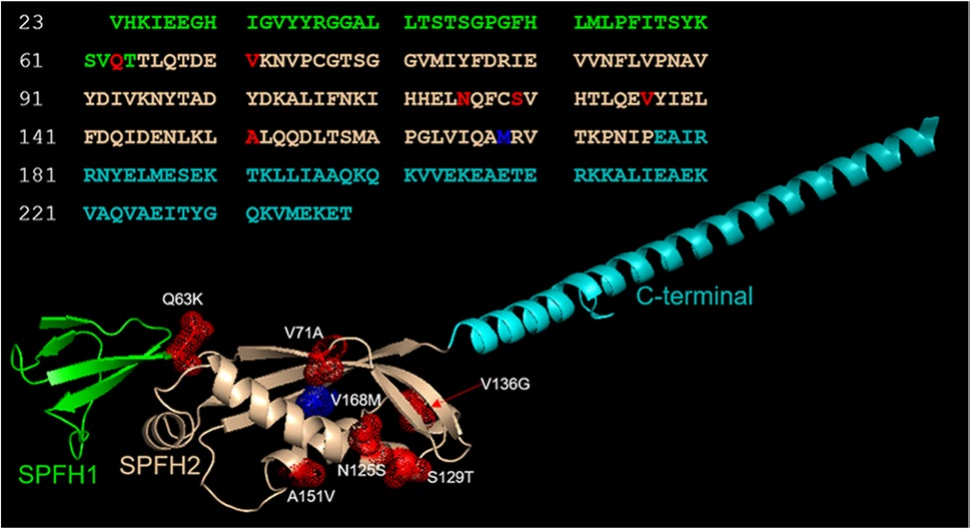
Analysis of erlin-2 structural model. Ribbon representation of the predicted structure of erlin2 (Val23-Thr238). Residues in the sequence are colored as the domains they belong to. Met168 is colored in blue, while the other residues involved in the mutations discussed in our analysis are colored in red. All mutated residues are mapped into the structure and represented as sticks and dots

Erlin2 is a 40 kDa transmembrane protein, localized at the endoplasmic reticulum (ER), belonging to the family of proteins containing the evolutionarily conserved SPFH (Stomatin/Prohibitin/Flotillin/HflK/C) domain [13]. To shed light on the molecular defect of V168M pathological mutant, the sequence of erlin2 (residues 23–238, Fig. 2) has been modeled using as a template the bacterial HflC protein structure [14]. The erlin2 structure is predicted to form two SPFH domains, a fold likely involved in cholesterol binding, followed by a C-terminal helix (Fig. 2).

Residue V168 is located in a β-strand of the SPFH2 domain where the substitution at position 168 of valine with methionine could perturb the fold, either by distortion due to the formation of a new H-bond [12] or by the increased steric hindrance of the side chain. Interestingly, the mapping of all dominant mutations reported by Chen et al. [12] in our model shows that they all fall within the SPFH domain (Fig. 2). Furthermore, a novel dominant mutation, V71A, recently reported in a Chinese family 5 maps in the SPFH domain, altogether suggesting a critical role of this region for the functions of erlin2.

Like the other members of the SPFH protein family, erlin2 displays high propensity to oligomerize by forming both homo- and hetero-oligomers with erlin1 as shown by co-immunoprecipitation assays of ectopically expressed erlin1 and 2 in different murine and human cell lines [15]. In the same study, the analysis of truncated and point mutants revealed an important role played by the C-terminal region in erlin2 oligomerization [15]. Thus, it is possible to envisage that the destabilization of the SPFH domain, due to missense mutations, could impact the orientation of the C-terminal region and consequently alters the formation of the homo- and hetero-dimers of erlin2.

**Fig. 3:**
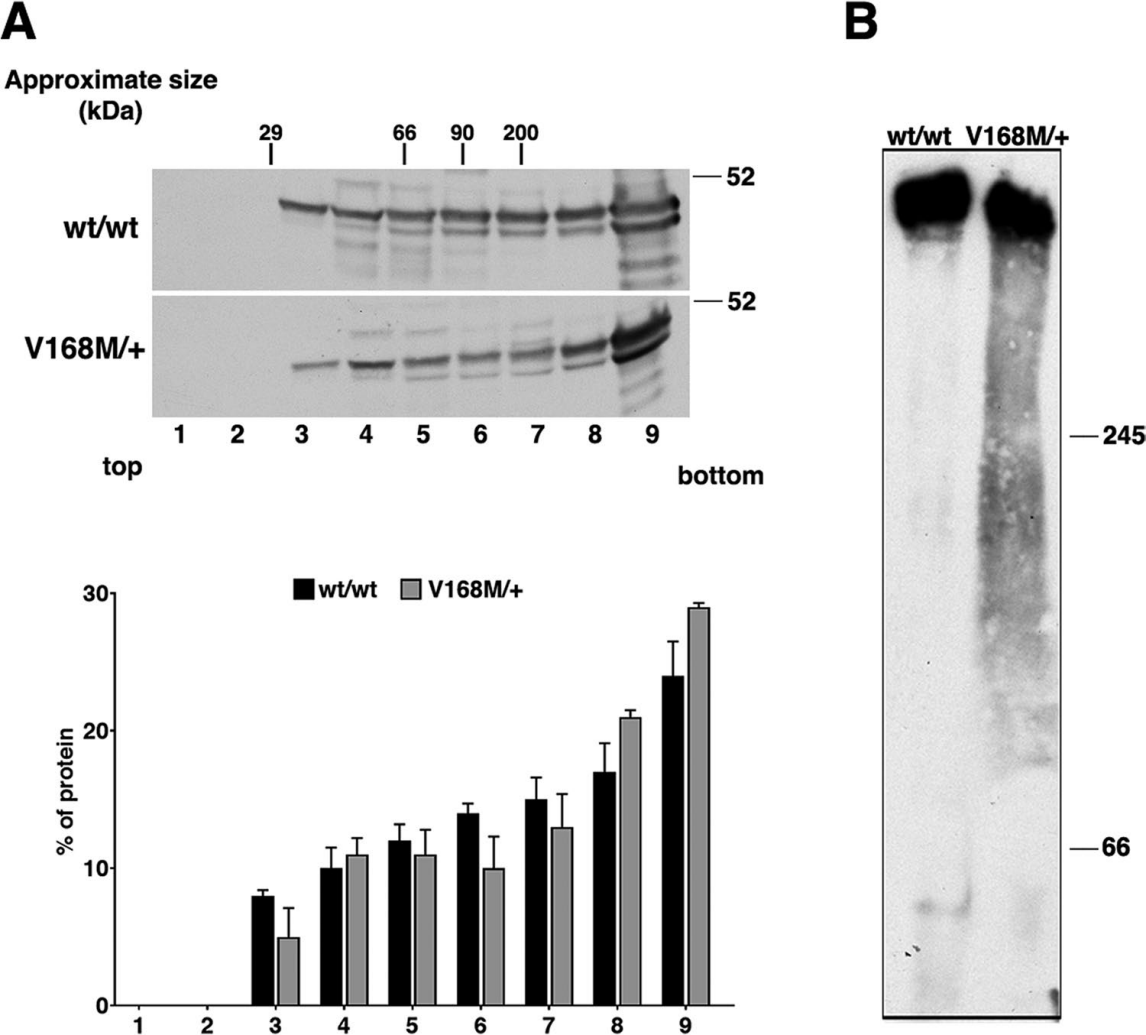
Analysis of oligomeric state of erlin2. The oligomeric state of erlin2 was evaluated by sedimentation on velocity gradient [8] (**A**) or by Blue native electrophoresis page [9] (BN-PAGE) (**B**). **A** Healthy subject and patient (III-12) fibroblasts were lysed in buffer containing 0.4% SDS and 0.2% Triton X-100 and run through 5–30% sucrose gradients. Fractions of 500 µl were collected from the top (fraction 1) to the bottom (fraction 9) of the gradients. Proteins were TCA-precipitated and detected by western blotting using a specific erlin2 antibody. The distribution on the gradients of the molecular mass markers is indicated. Densitometric analysis of protein distribution on the gradients, expressed as percentage in each fraction, is shown at the bottom; values are the mean of two different experiments ± S.D. **B** Healthy subject and patient (III-12) fibroblasts were homogenized in pounce homogenizer (30 strokes) in sucrose buffer (83 mM sucrose, 6.6 mM imidazole/HCL, pH 7.0) and centrifuged for 10 min at 20,000 g. Membrane pellets were extracted in the solubilization buffer (50 mM NaCl, 2 mM aminocaproic acid, 1 mM EDTA, 1% TX-100, 5% glycerol, pH 7.0). Protein samples (40 µg/lane) were resolved on a 6–15% gradient gel, then electroblotted to PVDF membrane and detected using a specific erlin2 antibody. Molecular weight standards are indicated

Therefore, we tested the ability of the mutated erlin2 to oligomerize in patient fibroblasts. In agreement with previous findings [15], in healthy control fibroblasts, erlin2 was purified as high molecular weight complexes (fractions 7–9 of the gradient) in addition to the gradient fractions corresponding to its expected monomeric molecular weights (Fig. 3A). Comparable distribution of erlin2 was observed in the patient fibroblasts (Fig. 3A), showing that both wild-type and V168M erlin2 are able to oligomerize. Consistently, erlin2 was isolated in native conditions as high molecular weight complexes similarly in control and patient fibroblasts by blue native polyacrylamide gel electrophoresis (BN-PAGE; Fig. 3B). Overall, these data indicate that V168M does not affect the ability of erlin2 to oligomerize.

## Discussion

The causal pathogenic mechanism of *ERLIN2* mutations in AR and AD HSP remains unknown. Currently, only a few HSP types, including SPG3A, SPG7, SPG9, SPG30, SPG58, and SPG72, have been shown to have both AD and AR patterns of inheritance; for them, a possible dominant negative effect and variable penetrance patterns have been postulated [2].

We describe the 20th SPG18 family. Up to now, 14 families have been reported with AR transmission and five with AD pattern of inheritance [1, 2, 4–6, 16–21]. While the AR kindred are associated with a complicated phenotype and an early onset, the AD ones show a pure HSP form and later onset.

Therefore, the erlin2 complete loss of function, most likely due to the AR mutations, seems to be responsible of a more severe and aggressive phenotype, while the dominant negative effect of the AD mutations is responsible of a milder disease and course.

We report that all the AD mutations fall in the SPFH domain strengthening the hypothesis of a dominant negative effect involving the interactions mediated by this specific domain. On the other hand, protein conformational modification due to mutations in SPFH domain could prevent erlin1/erlin2 complex. In this scenario, for the first time in human cells, we tested and excluded a possible dominant negative effect of V168M on erlin2 oligomerization. Since SPFH domain-containing proteins are found in lipid raft microdomains in diverse cellular membranes [13], it becomes important, at this point, to explore V168M impact on the membrane organization of erlin2.

Erlin2 is a mediator of ER-associated degradation (ERAD) pathway. For instance, erlin1/2 complexes regulate the proper turnover of inositol 1,4,5-trisphosphate calcium-channel receptors (IP3Rs), and a critical role of erlin-2 in binding IP3R is pointed out 22. Thus, it is likely that erlin2 deficiency may lead to abnormal neuronal signaling [23], [24]. However, we cannot exclude that the mutant forms of erlin2 may exert their pathogenetic mechanism impairing the degradation of other proteins. Moreover, persistent dysfunction of ERAD pathway may compromise ER homeostasis and functions. As consequence, the impairment of the plethora of ER-regulated pathways (such as protein quality control, trafficking, calcium homeostasis, lipid metabolism) and of the functional crosstalk with various cellular compartments (as mitochondria, Golgi, plasma membrane) may be hugely detrimental for cellular homeostasis.

Furthermore, erlin2 plays also a role in cholesterol metabolism by promoting ERAD of 3-hydroxy-3-methylglutaryl-coenzymeA (HMG-CoA) reductase (a key enzyme in the cholesterol synthesis) and by regulating sterol regulatory element binding protein (SREBP) signaling pathway [25, 26].

Thus, the biology of erlin2 underlies a really complex molecular scenario, implying that the alterations of different pathways can concur to the HSP’s pathogenesis on one side; to the other, it may also explain the clinical heterogeneity.

Of note, Ca ^2+^ homeostasis, lipid metabolism, mitochondrial biogenesis and trafficking, apoptosis, ER stress responses, autophagy, and inflammation are all implicated in neurodegeneration. Therefore, it may be important to monitor closely a possible evolution of HSP, in SPG18, not only in ALS but also in dementia.

ALS is also associated with fronto-temporal dementia (ALS/FTD) in 10% of cases [27]. Interestingly, a recent report describes a patient with SPG5 who secondly developed ALS/FTD at the age of 67 years, raising questions about a pathophysiological link between these conditions 28.

Moreover, ER plays an important role in maintenance of cellular proteostasis through three main mechanisms: the control of folding by chaperones, ER-associated degradation (ERAD), and the unfolded protein response (UPR). Of note, all of them are strongly deregulated in ALS, FTD, and Alzheimer’s disease [29, 30].

The four *ERLIN2* mutations, p.D69V, p.N125S, p.V168M, and p.D300V, up to now reported to show conversion in ALS [3, 6], apparently have no common features, either in term of their structural location, charge, or steric hindrance, and mode of inheritance. It is possible that the different spatial distribution of the mutations may have a different impact on protein activity, thus explaining the heterogeneity of the clinical phenotype. Functional studies are needed to clarify the mechanism that determines the onset of ALS.

Furthermore, the growing number of genes associated to allelic variant of HSP/ALS or HSP evolving to ALS, such as *CYP7B1*, *KIF5A*, *spatacsin*, *ERLIN1*, and *ALS2*, reiterate the question if these are two distinct entities or rather a spectrum of motor neuron involvement [31].

Surely, HSPs reveal a high level of phenotypic complexity presenting a large overlap with a variety of neurological phenotypes including not only ALS but also cerebellar ataxia, peripheral neuropathy, and epilepsy.

Like other genes involved in HSP complicated forms (mainly AR), *ERLIN2* is considered an epilepsy-associated gene [32].

We report the first AD family with generalized tonic–clonic epilepsy (pt IV-4), while Pt III-12 and Pt IV-11 had no epilepsy history but a clear pathologic EEG. To our knowledge seizures have rarely been described in the SPG18 spectrum [1, 4, 16, 17, 21, 33]. However, data are sometimes confusing and not very specific making difficult to identify the mutation linked to the epileptic phenotype [21, 33].

The first association between SPG18 and epilepsy was reported by Al-Yahyaee et al. in 2006 [16], who described two individuals with frequent epileptic seizures, normal brain CT scans, and EEG showing generalized epileptiform discharges.

Over the following years, Alazami et al. [17] described an AR family with two affected siblings carrying a nullimorphic *ERLIN2* mutation. Atypical absence epilepsy occurred in one sibling at the age of 7 years old, accompanied by a severely abnormal EEG and a normal MRI. Furthermore, myoclonic absence seizures were reported by Srivastava et al. [21]. Febrile convulsions were, instead, reported by Yildrim et al. in AR kindred with compound heterozygous truncating mutations [1].

Finally, Rydning et al. described an AD mutation in a patient with history of two childhood seizures and normal EEG, therefore, not defining a diagnosis of epilepsy [4].

Moreover, frontal EEG spikes and prefrontal EEG activity have been found to be effective in screening for dementia [34], while normal EEG makes the diagnosis of early dementia unlikely [35]. The presence of frontal epileptic activity (Pt.III-12) and the association to ALS in our family led us to speculate on the intrinsic association between SPG18 and FTD. Therefore, a close clinical and neuroradiological follow-up will be planned in order to evaluate the evolution in dementia.

In conclusion, we report the first SPG18 kindred with a complicated phenotype associated to an AD mode of inheritance.

Most of the clinical phenotypes (intellectual disability, epilepsy, ALS) described in SPG18 until today are resumed in our kindred revealing an interindividual and intrafamilial variability through generations despite the common genetic mutation. Indeed, p.V168M shows to have a high penetrance and variable expressivity and, consistently with a recent report, may convert to ASL around the age of 50 [3].

Taken together with molecular results, our data do not support the hypothesis of a tight relationship between the mode of inheritance and the phenotype and rule out a p.V168M dominant-negative effect on oligomerization. Fibroblasts from other patients with different dominant mutations will be very useful to delve deeper into the pathogenesis of SPG18 and to better understand erlin2 function.

The SPG18 spectrum is wider than we think, and a deeper clinical assessment is mandatory to better understand the disease. EEG should be always performed not only to screen for epilepsy but also in the prospective of FTD/ALS because of its high specific value in the context of the diagnosis of early dementia.

## Data Availability

The data that support the findings of this study are not openly available due to reasons of sensitivity/privacy and are available from the corresponding author (C.C.) upon reasonable request.
